# Exploring the relationship between health behavior and sleep quality: *preliminary* insights from ECG-derived sleep analysis

**DOI:** 10.1007/s00702-025-03046-3

**Published:** 2025-10-14

**Authors:** Claudia Traunmüller, Andreas R. Schwerdtfeger

**Affiliations:** 1 Institute of Psychology, University of Graz, 8010 Graz, Austria; 2 Health Psychology Unit, Department of Psychology, University of Graz, Universitaetsplatz 2/III, Graz, 8010 Austria

**Keywords:** Health behavior, Sleep quality, Sleep stages, SleepECG algorithm

## Abstract

Correlations between sleep quality, health behavior, and metabolic variables are empirically well documented. This study investigated a novel algorithm to determine sleep stages by leveraging ECG data. The aim was to examine whether this algorithm effectively capture correlations with health behavior, self-reported sleep quality, and metabolic variables. A cross sectional correlation study design was used to survey health behavior of a total of 194 healthy individuals (87 female; mean age, 40.29 ± 11.8) with a focus on BMI, physical activity, subjective sleep quality via the PSQI and eating habits (daily fruit and vegetable consumption). In addition, a 24 h ECG was derived to determine sleep stages. Positive associations were found between sleep stages, sleep duration and sleep quality. Moderate physical activity and fruit and vegetable consumption were positively associated with sleep stages. Negative correlations were found between BMI and sleep stages. Overall, health behavior variables could predict duration of NREM (*R*^2^ = 0.040, *F*(4/186) = 2.98, *p* < .021) and REM (*R*^2^ = 0.050, *F*(4/188) = 3.50, *p* < .009), as well as numbers of NREM and REM episodes (*R*^2^ = 0.065, *F*(4/186) = 4.30, *p* < .002; *R*^2^ = 0.077, *F*(4/186) = 4.99, *p* < .001). However, health behavior variables could not predict sleep duration (*R*^2^ *=* 0.010, *F*(4/168) = 1,44, *p* < .222). The SleepECG algorithm supported correlations between health behaviors, BMI, and self-reported sleep quality, indicating its potential as a practical and cost-effective method for objectively measuring sleep when polysomnography is not feasible.

## Introduction

Sleep plays a vital role in human health and is essential for sustaining physical, emotional and cognitive wellbeing (Rechtschaffen and Bergmann 2002; Hirshkowitz et al. 2015). With a growing number of individuals expressing concerns about poor sleep quality and its impact on daily functioning, sleep has become an increasingly significant target for research. A comprehensive review encompassing numerous epidemiological studies revealed that nearly one third of the general population are affected by sleep problems like symptoms of insomnia (defined as difficulties falling asleep), while approximately 4% and 26% report excessive sleepiness (Ohayon 2011) and around 17% of adults suffer from accompanying mental health problems (Bebbington and McManus 2020). Compelling evidence underscores the influence of sleep quantity and quality on human health (Godos et al. 2021). Insufficient sleep duration and/or inadequate sleep quality has been associated with elevated risk of cardiovascular disease (Cappuccio et al. 2011), diabetes (Cappuccio et al. 2010), cancer (Chen et al. 2018), as well as incidence of depression (Wang and Boros 2021) and mental disorders (Bebbington and McManus 2020). Correspondingly, it has been convincingly outvoiced to consider sleep an important health behavior (Chaput and Dutil 2016).

Noteworthy, extensive evidence links health behavior to sleep (Prendergast et al. 2016; Conner et al. 2017; Raboch et al. 2017; Nho and Yoo 2018; Francis et al. 2019; Chin et al. 2019; Wickham et al. 2020). Healthy lifestyle encompasses various behaviors, including physical activity (PA) and healthy food intake (Tan et al. 2018). For example, Du et al (2024) reported associations between sleep duration, poor sleep quality, unhealthy diet, and physical inactivity. Consequently, the World Health Organization has emphasized the importance of physical activity and fruit/vegetable intake as major targets for health promotion (Mendis et al. 2015). Importantly, defining sleep quality and the measure used to characterize poor or good sleep quality is crucial. A “good sleep” is a night with a sufficient sleep duration that provides enough time for the homeostatic restorative process (Barbato 2021). Although sleep duration is the most extensively studied and robustly supported measure in examining its relationship to health (Watson et al. 2015), it is increasingly evident that sleep quality rather than sleep quantity appears to be a central predictor of mental health and well-being among the general population (Pilcher et al. 1997; Bassett et al. 2015; Wallace et al. 2017; João et al. 2018), particularly among young adults (Buysse et al. 2008).

### Sleep quality and physical activity

Modifiable risk factors such as PA may contribute to improve sleep quality (Greever et al. 2017). Thus, understanding the association between PA and sleep quality is crucial, with a special focus on precise classification of PA intensity, indicating the level of exertion during the activity. Cross sectional studies have demonstrated that active adolescents experienced more favorable sleep quality as compared to their inactive counterparts (King et al. 2008b, Bisson et al. 2019). The intensity of activity seems to play a significant role in this relationship, suggesting that increased PA, especially moderate to vigorous PA, is a better predictor for good sleep as compared to low PA (King et al. 2008a; Wang and Boros 2021).

### Sleep quality and diet

Recent reviews of the literature provide interesting insights into the potential association between diet and sleep (Peuhkuri et al. 2012; St-Onge et al. 2016). Noorwali et al. (2018) investigated the relation between sleep and healthy diet and revealed a positive association between the amount of fruit and vegetable intake and sleep quality. Sleep quality was assessed by self-reported sleep duration, and the consumption of fruits/vegetables was assessed by a 4-day food diary. Results could be replicated by Ferranti et al. (2016), where good sleep quality was also positively associated with higher fruit and vegetable consumption. In line with that, an online survey study also found positive correlations between fruit and vegetable intake and sleep quality (Chaput and Dutil 2016). Notable, due to cross-sectional study designs, these studies cannot address causality or the direction of the relation between variables (St-Onge et al. 2016).

### Sleep quality and metabolic status

Associations between metabolic status and both duration and quality of sleep have been investigated extensively in cross-sectional and longitudinal studies in the general adult population (Knutson 2012; Nagai et al. 2013; Moraes et al. 2013; Gutiérrez-Repiso et al. 2014). Both negative linear correlations and U-shaped relationships were observed between sleep duration and body mass index (BMI), depending on age and the length of sleep (short < 5 h versus long > 8 h), suggesting that high levels of BMI are accompanied by poorer sleep.

### Subjective methods of sleep quality assessment

Subjective reports of sleep quality play a crucial role in identifying the need for further screening and treatment of sleep complaints (Åkerstedt et al. 2002). Among the subjective measures, the sleep diary is the most widely-used assessment tool (Natale et al. 2015), asking individuals to provide morning ratings regarding various aspects of their sleep. Among sleep questionnaires, the Pittsburgh Sleep Quality Index (PSQI, Buysse et al. 1989) is the most widely used measure of subjective self-reported sleep quality, which provides a measure of global sleep quality based on respondent´s retrospective appraisal of an array of sleep indices, including sleep latency, sleep duration, habitual sleep efficiency, sleep disturbances, use of sleep medication, and day time dysfunction (Buysse et al. 1989).

### Objective methods of sleep quality assessment

By using objective methods like polysomnography (PSG), the gold standard to objectively evaluate sleep, sleep quality can be defined by sleep latency, sleep duration, sleep efficiency, sleep stages (i.e. stages 1–4 and REM sleep) (Krystal and Edinger 2008), and sleep pattern (Fabbri et al. 2021). Total sleep time appears to be one of the most reliable objective proxy measures of sleep quality (Barbato 2021), although sleep quality encompasses not only the total duration of sleep, but also its architecture, defined by the amount of different sleep stages across the sleep episode (O’Donnell et al. 2009). The human body typically oscillates between two main phases of sleep: rapid eye movement (REM) and non-rapid eye movement (NREM) sleep, the latter being further categorized into three substages (N1 - N3), each leading to progressively deeper sleep (Malik et al. 2018). NREM sleep is considered to be restorative and recuperative for the body and accounts typically 75–80% of total sleep time (Gunning 2001), while REM sleep, which is associated with dreaming, accounts for around 20–25% of total sleep time (Carskadon and Dement 2005).

Of note, analysis of sleep stages in different age groups suggested associations between subjective sleep quality and elevated duration of both REM (O’Donnell et al. 2009) and NREM sleep (Westerlund et al. 2016). Moreover, an increase of overnight sleep that is staged as REM had been associated with better sleep quality (Wang et al. 2013). Taken together, a detailed analysis of sleep stages could be useful for evaluating the effects of health behaviors on human sleep. Unfortunately, since PSG is expensive, demanding for the individual and hence, not available for everybody, developing alternative approaches to quantify sleep stages applicable in everyday life seems mandatory. Specifically, analysis of.

cardiac activity during sleep could constitute a viable option to assess sleep stages as a proxy measure of (objective) sleep quality, thereby reducing costs and individual burden (Agnew et al. 1967).

This study aimed to explore the validity of a novel algorithm utilizing machine learning techniques to determine sleep stages (REM and NREM sleep scoring) by leveraging ECG data assessed in everyday life (for further details see Brunner and Hofer (2023). The goal was to conduct a first test of this algorithm to verify correlations between health behavior and objectively measured sleep quality via ECG signal. We hypothesized that NREM and REM episodes derived from ECG sleep analysis would be positively associated with subjective sleep quality, physical activity, healthy diet, and negatively with BMI.

## Materials and methods

We used a cross-sectional correlational design with data collected through an online survey combined with ambulatory assessment of ECG data. In total, 194 individuals (87 female; mean age, 40.29 ± 11.8) participated in the study working within a big company in Austria.

### Procedure

The study was part of a larger project aiming to validate a newly developed Health Behavior Questionnaire. Prior to the commencement of the study, a comprehensive video podcast was recorded in collaboration with the company’s executive board. This podcast provided a detailed explanation and information of the study, including its complete structure, information on the collected variables, and a particular emphasis on data security and the benefits for the participants. The advantage of the podcast was to personally inform all employees of the company about the planned project, thereby eliminating the need to individually explain the project to each person during the recruitment process.

The link to this podcast was disseminated to all employees by the Human Resources department via email. Furthermore, these details were made accessible on the internal company intranet. One week after the distribution of the podcast, the recruitment process started. Recruitment and data collection was scheduled during April and July of 2024.

### Participants

To mitigate recruitment bias, 200 adult individuals (ages > 18 years) within the company were recruited using a randomization process. The Human Resources department of the company provided a list containing the work phone numbers of all employees, accompanied by additional information including age, gender, department affiliation, and the respective location at which each individual worked. Notably, for data protection reasons, the names of the individuals were not included in this list. Following this, employees were randomly contacted via phone and invited to participate in the study. Data from individuals of all departments were collected to obtain a representative average within the company. It was explicitly communicated to them that participation was voluntary and that they could discontinue participation any time without providing a reason. Upon agreement, each participant was assigned a unique code. Furthermore, participants were emailed a consent form to be signed and returned to us via email. Subsequently, all participants received a link to an online test battery, through which they could log in using their assigned code. This test battery included a socio-demographic questionnaire and an assessment of current health behaviors across multiple dimensions of health. Individuals who did not have acute mental illnesses and had not experienced any cardiovascular diseases within the last year were eligible to participate in the study. In the end, 194 individuals were successfully recruited. For descriptive statistics related to the sample, refer to Table 1. All procedures performed in studies involving human participants were in accordance with the ethical standards of the institutional and/or national research committee (GZ. 39/7/63 ex 2013/14) and with the 1964 Helsinki declaration and its later amendments or comparable ethical standards.

**Table 1:**
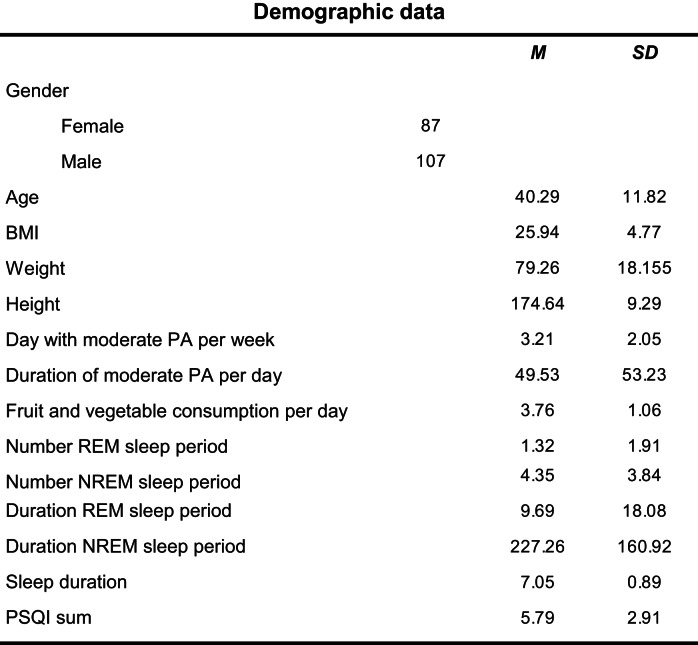
Demographic data, sleep stages, sleep duration and sleep quality

*Weight* kg, *height* cm, *PA* physical activity, *REM* Rapid Eye Movment, *NREM* Non Rapdi Eye Movement

### Study procedure

Upon consenting to participate in the project during the initial phone contact, each participant was scheduled for three appointments at our laboratory. At the first appointment, scheduled between 6am and 8am, each participant was fitted with a 24-hour ECG recorder (eMotion Faros 180^®^; Bittium, Oulu, Finland) with a sampling rate of 500 Hz to be worn continuously in order to be able to determine sleep stages The ECG recorder was then removed during the second appointment, scheduled for the following day at the same time, where also BMI was assessed.

#### Measure of health behavior

Sleep quality and quantity: *The Health Behavior Questionnaire basically contains several questions related to sleep*,* such as what time on average individuals get up and go to bed*,* how often they wake up during the night*,* and how they feel the following morning. Based on previous research related to the association between self-reported sleep duration and health consequences (*Gupta et al. 2002; Hasler et al. 2004; Gangwisch et al. 2005; Gong et al. 2017), *in this study*,* however*,* we were only interested in sleep duration*,* which is why only one adapted single item of the* Pittsburgh Sleep Quality Index (PSQI, Buysse et al. (1989) was used: “How many hours do you usually sleep per night?. Original Item: “During the past month, how many hours of actual sleep did you get at night? Furthermore, sleep quality was quantified by calculating the sum score of the PSQI.

Additionally; each participant underwent a 24-hour ECG monitoring, during which they documented sleep onset and awakening time. These data were analyzed using the Sleep ECG algorithm. *SleepECG is an open-source Python package for sleep staging based on heart rate variability (HRV) derived from ECG signals (*Brunner and Hofer 2023*). The pipeline comprises the following main steps: heartbeat detection using an optimized implementation of the* Pan and Tompkins (1985*) extraction of up to 26 time-domain and 7 frequency-domain HRV features (including successive differences*,* Poincaré plots*,* and power spectral density)(*Task Force of the European Society of Cardiology and the North American Society of Pacing and Electrophysiology 1996; Shaffer and Ginsberg 2017*) and sleep stage classification with pre-trained gated recurrent unit networks (implemented with TensorFlow)*,* achieving 83% accuracy for wake/sleep and 75% for wake/REM/NREM classification on unseen test data. A core principle of SleepECG is reproducibility. The package integrates with large*,* publicly available datasets (e.g.*,* MESA*,* SHHS*,* MIT-BIH) and provides tools for downloading*,* preprocessing*,* and analysis. The entire pipeline*,* including classifier training*,* is open source (*https://github.com/cbrnr/sleepecg*)*,* and all datasets are publicly available*,* although some require registration through the National Sleep Research Resource (NSRR) website (*https://sleepdata.org/*).*

PA: The amount and intensity of PA within a week was assessed by following questions, adapted from the International Physical Activity Questionnaire (Maddison et al. 2007): “How many days a week do you do strenuous physical activity (e.g. aerobics, running, fast cycling, swimming)? “How much time did this physical activity take per day (in minutes)?”,” How many days a week do you do light to moderate physical activity (e.g., easy walking, easy cycling)? and “How much time did this physical activity take per day (in minutes)?”. The variables ‘number moderate/vigorous PA per week’ and ‘duration moderate/vigorous PA per day’ were calculated from these questions.

Eating behavior: Participants were asked to rate their typical consumption of fresh fruits and vegetables in a day via the following question: “Do you eat fresh fruit and vegetables every day? Participants had to rate this on a 5-point scale from 1 (never) to 5 (very often).

### Statistical analysis

Statistical analysis was conducted using the software IBM SPSS statistics version 29 (IBM Corp., NY, USA) by calculating Pearson correlations and multiple regression analyses. To support the assumption of normally distributed data, a Shapiro Wilk Test was conducted. Examining heteroscedasticity, a Breusch-Pagan Test was conducted. Results indicated no significant heteroscedasticity, X²(1) = 0.671, *p* = .413. To assess autocorrelation in the residuals, a Durbin-Watson Test was utilized. The test yielded a value of 1.902, which is within the acceptable range, indicating no significant autocorrelation. Multicollinearity was assessed using Variance Inflation Factor (VIF) analysis. All VIF values were below the commonly accepted threshold of 10, indicating that multicollinearity was not a concern in the current model. Subsequently, the relationship between sleep quality, health behavior variables, and Body Mass Index (BMI) was assessed through regression analyses. In addition, linear regression models were conducted to examine the associations between various predictor variables, such as sleep duration, Rapid Eye Movement (REM) and Non-Rapid Eye Movement (NREM) sleep periods, and the independent variables. The general level of significance was fixed at *p* < .05 (two tailed) for all analysis.

## Results

Pearson correlations among variables are depicted in Table 2.

**Table 2:** Correlations between sleep duration, sleep stages, health behavior and BMI

	BMI	Number moderate PA per week	Duration moderate PA per day	Number vigorous PA per week	Duration vigorous PA per day	Fruit and vegetable per day	Sleep Duration	REM Number	NREM Number	REM Duration	NREM Duration	PSQI sum
BMI												
r	1.000	-0.134	-0.003	-0.162^*^	-0.151^*^	-0.161^*^	0.010	-0.157^*^	-0.043	-0.205^**^	-0.121	0.160
p		0.063	0.964	0.025	0.036	0.026	0.890	0.029	0.555	0.004	0.093	0.080
N	196	193	193	193	193	193	176	194	194	196	194	121
Number moderate PA per week												
r	-0.134	1.000	0.106	0.227^**^	0.129	0.118	0.103	-0.020	0.029	0.015	0.057	0.055
p	0.063		0.143	0.001	0.072	0.102	0.177	0.781	0.687	0.834	0.431	0.551
N	193	194	194	194	194	194	174	192	192	194	192	121
Duration moderate PA per day												
r	-0.003	0.106	1.000	0.123	0.250^**^	0.008	0.067	0.245^**^	0.215^**^	0.257^**^	0.094	-0.142
p	0.964	0.143		0.087	0.000	0.917	0.380	0.001	0.003	0.000	0.196	0.121
N	193	194	194	194	194	194	174	192	192	194	192	121
Number vigorous PA per week												
r	-0.162^*^	0.227^**^	0.123	1.000	0.729^**^	0.158^*^	0.101	0.002	0.113	-0.028	0.044	0.058
p	0.025	0.001	0.087		0.000	0.028	0.186	0.974	0.119	0.703	0.548	0.527
N	193	194	194	194	194	194	174	192	192	194	192	121
Duration vigorous PA per day												
r	-0.151^*^	0.129	0.250^**^	0.729^**^	1.000	0.119	0.062	-0.012	0.081	-0.031	0.015	-0.064
p	0.036	0.072	0.000	0.000		0.098	0.418	0.870	0.264	0.667	0.833	0.484
N	193	194	194	194	194	194	174	192	192	194	192	121
Fruit and vegetable per day												
r	-0.161^*^	0.118	0.008	0.158^*^	0.119	1.000	0.150^*^	0.168^*^	0.205^**^	0.154^*^	0.171^*^	-0.197^*^
p	0.026	0.102	0.917	0.028	0.098		0.048	0.020	0.004	0.032	0.018	0.030
N	193	194	194	194	194	194	174	192	192	194	192	121
Sleep Duration												
r	0.010	0.103	0.067	0.101	0.062	0.150^*^						
p	0.890	0.177	0.380	0.186	0.418	0.048						
N	176	174	174	174	174	174	177					
REM Number												
r	-0.157^*^	-0.020	0.245^**^	0.002	-0.012	0.168^*^	0.254^**^		^**^			
p	0.029	0.781	0.001	0.974	0.870	0.020	0.001					
N	194	192	192	192	192	192	176	203				
NREM Number												
r	-0.043	0.029	0.215^**^	0.113	0.081	0.205^**^	0.257^**^	0.653^**^				
p	0.555	0.687	0.003	0.119	0.264	0.004	0.001	0.000				
N	194	192	192	192	192	192	176	203	203			
REM Duration												
r	-0.205^**^	0.015	0.257^**^	-0.028	-0.031	0.154^*^	0.179^*^	0.937^**^	0.583^**^			
p	0.004	0.834	0.000	0.703	0.667	0.032	0.017	0.000	0.000			
N	196	194	194	194	194	194	177	203	203	205		
NREM Duration												
r	-0.121	0.057	0.094	0.044	0.015	0.171^*^	0.351^**^	0.582^**^	0.594^**^	0.549^**^		
p	0.093	0.431	0.196	0.548	0.833	0.018	0.000	0.000	0.000	0.000		
N	194	192	192	192	192	192	176	203	203	203	203	
PSQI_sum												
r	0.160	0.055	-0.142	0.058	-0.064	-0.197^*^	-0.047	-0.182^*^	-0.255^**^	-0.166	-0.053	
p	0.080	0.551	0.121	0.527	0.484	0.030	0.624	0.046	0.005	0.068	0.563	
N	121	121	121	121	121	121	113	121	121	121	121	121

*PA* physical activity, *REM* Numbers of Rapid Eye Movement periods, *NREM* Numbers of Non-Rapid Eye Movement periods,* PSQI sum* Sum Score of the Pittsburgh Sleep Quality Index

*p < .05, **p < .01, ***p < .001

### Associations between sleep duration and sleep stages

Significant positive correlations between sleep duration and the number and duration of REM periods (*r* = .254, *p* < .001; *r* = .179, *p* = .017) and the number and duration of NREM periods (*r* = 257, *p* < .001; *r* = .351, *p* < .001) were found, indicating that an increase in sleep duration was associated with an increase in both REM and NREM episodes and length. Additionally, we found a negative correlation between the sum score of the PSQI and the numbers of REM and NREM periods, respectively (*r* = − .182, *p* = .046; *r* = − .255, *p* = .005). These findings document that the better the sleep quality the higher the numbers of REM and NREM periods was (of note, the higher the PSQI sum score, the worse the quality of sleep). No significant correlation was found between PSQI and sleep duration.

### Associations between sleep quality and PA

As the quality of sleep might be influenced by the intensity of PA (Semplonius & Willoughby 2018), we calculated associations with the different intensities of PA separately. Associations could be found between moderate PA and sleep stages. Specifically, there were significant positive correlations between the duration of moderate PA (measured in minutes) and both the number and duration of REM periods (*r* = .245, *p* < .001, *r* = .257, *p* < .001), as well as the number of NREM periods (*r* = .215, *p* = .003). However, no significant correlations were found between moderate PA and sleep duration and between vigorous PA and subjective/objective measured indicators of sleep quality (Table 1).

### Associations between sleep quality and diet

We found positive correlations between the amount of fruit and vegetable consumption per day and both objective and subjective sleep variables. In particular, fruit and vegetable consumption were significantly positively correlated with sleep duration (*r* = .150, *p* = .048), number and duration of REM periods (*r* = .168, *p* = .020; *r* = .154, *p* = .032), and number and duration of NREM periods (*r* = 205, *p* = .004; *r* = .171, *p* = .018). Moreover, we found a negative correlation between the sum score of PSQI and the diet variable (*r* = − .197, *p* = .030).

### Association between sleep quality and BMI

Negative correlations could be found between BMI and the number and duration of REM periods (*r* = − .157, *p* = .029; *r* = − .205, *p* = .004). No significant correlations could be found between sleep duration, PSQI, NREM periods and BMI, respectively.

### Linear regression analyses

Subsequently, multiple regression analyses were conducted to further examine these relationships in concert and to identify the predictive power of the selected variables in determining sleep quality outcomes. BMI, daily fruit and vegetables consumption, frequency and duration of moderate physical activity per week served as predictor variables, and sleep duration, number and duration of REM- and NREM sleep periods as criterion variables.

The first regression analysis for sleep duration indicated no significant prediction (*R*^2^ *= 0.010*,* F*(4/168) = 1.44, *p* < .222), thus suggesting that none of the predictor variables were meaningful. Subsequent analysis for the duration of NREM sleep as criterion variable showed significant results (*R*^2^ = 0.040, *F*(4/186) = 2.98, *p* = .021). Of note, BMI (*β* = − 0.144, *t*(186) = -2.00, *p* = .047) and daily fruit and vegetables consumption (β = 0.172,, *t*(186) = 2.39, *p* = .018) could significantly predict NREM duration. The model for predicting the number of NREM sleep episodes was also significant (*R*^2^ = 0.065, *F*(4/186) = 4.30, *p =* .002). Both PA (*β* = − 0.176, *t*(186) = 2.47, *p* = .005) and the consumption of fruit and vegetables (*β* = 0.204, *t*(186) = 2.87, *p* = .005) served as independent significant predictors (Table 3). The model for predicting REM duration was also significant (*R*^2^ = 0.050, *F*(4/188) = 3.50, *p* = .009). Notably, only BMI (*β* = − 0.203, *t*(188) = -2.85, *p* = .005) turned out as a significant predictor. Lastly, analysis for the number of REM sleep episodes indicated significant results (*R*^2^ = 0.077, *F*(4/186), *p* < .001) with almost all predictor variables being significantly associated with the outcome. Except for BMI (*β* = − 0.137, *t*(186) = -1.95, *p* = .053) and physical activity (*β* = − 0.137, *t*(186) = -1.93, *p* = .056), which were marginally significant, fruit and vegetable consumption (*β* = 0.182, *t*(186) = 2.57, *p*.

**Table 3:**
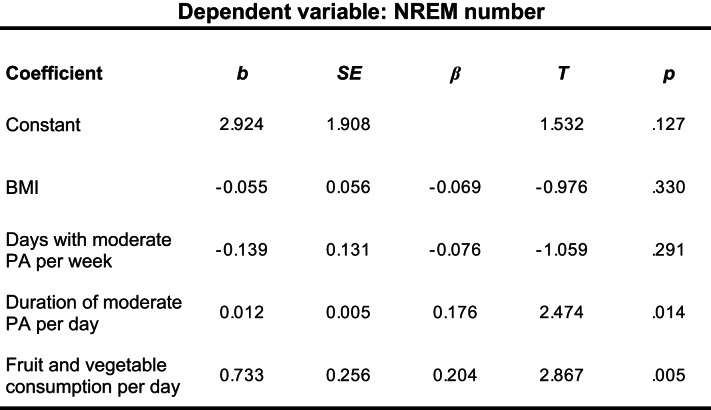
Results of a multiple regression analysis of NREM numbers on BMI, PA, and fruit and vegetable consumption

*N* = 186, *R*^2^ = 0.085, adj. *R*^2^ = 0.065, *F*(4, 186) = 4.298, *p* = .002

< 0.001) and the duration of physical activity (*β* = 0.173, *t*(186) = 2,46, *p* = .015) were significantly related to the number of REM sleep periods (Table 4).

**Table 4:**
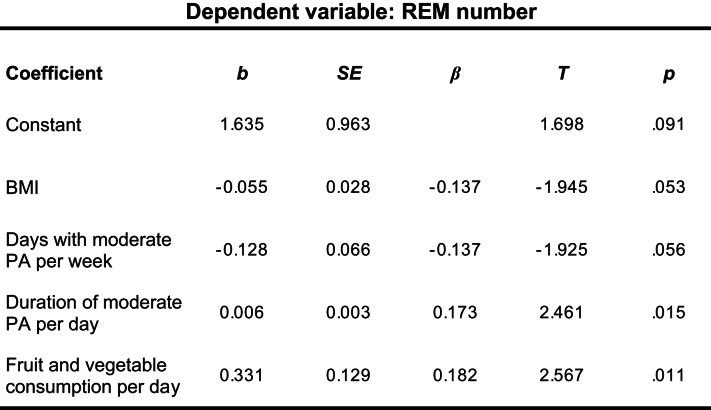
Results of a multiple regression analysis of REM numbers on BMI, PA, and fruit and vegetable consumption

*N* = 186, *R*^2^ = 0.097, adj. *R*^2^ = 0.077, *F*(4, 186) = 4.988, *p* = .001

## Discussion

The aim of this study was to evaluate the performance of a SleepECG algorithm (Brunner and Hofer 2023) to replicate correlations between health behaviors and sleep quality as shown in previous research (Conner et al. 2017; Francis et al. 2019; Chin et al. 2019). In order to accomplish this aim, different lifestyle factors were assessed from 194 healthy individuals and subsequently correlated with both subjective (sleep duration) and objective (SleepECG) sleep quality. In light of evidence of a significant correlation between sleep duration and sleep stages (O’Donnell et al., 2009; Westerlund et al. 2016), we first evaluated this correlation using the SleepECG algorithm. Results confirmed a significant association between sleep duration and both REM and NREM sleep stages. Moreover, moderate PA and the sum score of the PSQI were correlated with sleep stages but not with sleep duration. Furthermore, positive correlations could be found between fruit and vegetable consumption and both subjective and objective sleep quality variables. Negative correlations could be shown for BMI and sleep stages, but not for sleep duration. Importantly, it could be demonstrated that health behavior variables could predict sleep stages but not sleep duration.

Sleep duration is seen as a reliable estimate of sleep quality (Barbato 2021), although this parameter cannot provide additional information apart from hours slept. Importantly, sleep stages fulfill different functions that have a major impact on our health. While REM stage is essential for processing emotional and complex information of the day (Wang et al. 2013), NREM stage is seen as the most restorative sleep stage, facilitating physical recovery, the strengthening of the immune system, and the growth and repair tissues. Moreover, NREM seems fundamental for cognitive functions (Gunning 2001). Hence, both REM and NREM sleep serve mental health and should be considered when evaluating the relationship between sleep and psychological and behavioral variables. The applied SleepECG algorithm seemed suitable to quantify REM and NREM episodes in daily nocturnal ECG recordings, which offers a worthwhile, low-threshold approach to collect more nuanced data on sleep in ambulatory contexts. *It is important to mention that this study is based on a specific sample. This means that the results cannot be generalized. Further research with different samples in terms of stress profiles and diverse demographics are needed in order to replicate the results and thus be able to generalize them.*

The significant association between sleep stages and moderate PA corroborate previous research on this topic (Sherrill et al. 1998; Kredlow et al. 2015; Seol et al. 2022). An increase in moderate PA correlates positively with REM and NREM stages, which signifies better sleep quality (Wang et al. 2013). Semplonius and Willoughby (2018) also reported that moderate PA indirectly predicted sleep quality over time via a positive effect on emotional regulation. This is in line with previous findings of Bisson et al. (2019), who reported low to moderate PA to be related with better sleep quality but not sleep duration. Moreover, our analyses did not find any significant correlation between vigorous PA sleep quality measures. These results were confirmed by a review article (Wang and Boros 2021), which found that moderate PA seems to be more effective than vigorous activity in improving sleep quality.

*Another possible explanation for the effects of different intensities of PA on sleep quality could be that participants found it difficult to distinguish precisely between moderate and intense physical activity when answering the questionnaire*,* although the different intensities were explained. It should be also noted that in the present study*,* only one third of the respondents stated that they exercised at a vigorous intensity level. Due to the limited proportion of the sample engaging in vigorous activity*,* the power for respective findings could have been too low. Future studies should address these issues by incorporating objective measures of physical activity*,* such as accelerometers*,* to enable a more accurate distinction between activity intensities and their potential effects on sleep stages.*

*An additional explanation for the influence of different intensities of PA on sleep quality could lie in the time at which the PA was performed*,* which was not assessed in our study. A systematic review published by* Stutz et al. (2019) *showed that both the time prior to sleep and intensity of PA have an impact on sleep quality. It was demonstrated that PA in the evening generally had a positive effect on sleep quality*,* while vigorous physical activity in the evening had a negative impact on sleep quality. This in in line with a study of* Oda and Shirakawa (2014*) who also pointed out that vigorous exercise right before bedtime can have a negative impact on sleep quality*,* caused by a large physiologic excitement prior to going asleep. The authors conclude that for moderately fit individuals a longer recovery period could be beneficial. Importantly*,* in further studies the intensity and the timing of PA (i.e. close to bedtime) need to be taken into account when analyzing the relationship between PA and sleep quality. These results suggest that “the more the better” is not always true and that engaging in moderate PA may especially benefit sleep quality*,* which is of practical importance for promoting health.*

Dietary habits, particularly fruit and vegetable consumption, was significantly correlated with various sleep quality indicators, like sleep duration and sleep stages. This finding is consistent with literature emphasizing nutrition’s role in modulating sleep health (Peuhkuri et al. 2012; St-Onge et al. 2016). Peuhkuri et al. (2012), for example, could observe that those individuals, who sleep less were more likely to consume energy-rich food and fewer portions of fruits and vegetables. Whether ingested food directly affects sleep is difficult to address, however, results could show that a balanced and varied diet that is rich in fresh fruits and vegetables can improve sleep quality. This is in line with the results of a review article by Onge et al. (2016) emphasizing the sleep promoting effect of fruit and vegetable consumption. Furthermore, Godos et al., (2016) showed that the consumption of healthy food like fruits and vegetables was associated with better sleep quality, whereas higher intake of processed and free-sugar rich food was associated with worse sleep quality. These findings could guide dietary interventions aimed at improving sleep quality.

The relationship between BMI and sleep quality turned out to be negative, as expected from previous research (Vargas et al. 2014; Krističević et al. 2018; Wang et al. 2019). Results were quite fragile though, revealing a decrease in REM- but not in NREM sleep stages or sleep duration. *One explanation for these results could be the association between autonomic nervous system (ANS) dysfunction and obesity (*Gupta et al. 2002; Baum et al. 2013; Guarino et al. 2017). *In particular*,* it has been suggested that an elevated BMI is often associated with chronic activity of the sympathetic nervous system*,* which could affect the regulation of REM sleep (*Landsberg and Krieger 1989; Guarino et al. 2017). Landsberg and Krieger (1989*) also pointed out that chronic hyperinsulinemia*,* a characteristic of obesity*,* can further increase sympathetic activity*,* which might negatively affect REM sleep architecture.*

Guarino et al. (2017) *offer another possible explanation and point to the link between increased BMI and mild chronic inflammation*,* caused by the release of cytokines (e.g. TNF-α*,* IL-6) from visceral adipose tissue. According to the authors*,* this inflammation can influence the regulation of the central nervous system and subsequently impair REM sleep quality. Another explanation could lie in the metabolic consequences of obesity and its effects of sleep as research of* Van Cauter et al. (2008) *has shown. Leptin resistance (despite elevated leptin levels) and elevated ghrelin levels can disrupt the sleep-wake cycle*,* with these hormonal changes particularly affecting the REM phase*,* as they are closely linked to the regulation of the sympathetic nervous system. The authors also emphasize that although NREM sleep plays an important role in metabolic regulation*,* it is less sensitive to metabolic and autonomic changes in obesity than REM sleep.*

Overall, the present results argue for a potential benefit of incorporating more fine-grained measures of sleep quality in future research to better understand the complex relationship between being overweight and sleep quality.

### Limitations

Although this study suggests the usefulness and validity of applying ECG analysis to derive sleep stages in everyday life recordings, there are several limitations that need to be mentioned. First, although our results provide valuable insights into the relationships between health behaviors and sleep quality, future studies with longitudinal or experimental designs are needed to establish causal relationships. Second, the generally small effect sizes of the associations observed challenge the delineation of direct practical or clinical implications. Third, the validation study of the SleepECG-algorithm revealed a limited specificity of approximately 78% in correctly classifying sleep variables (Brunner and Hofer 2023), indicating the need for further refinement (e.g., by adding accelerometer data), which in turn, could yield larger effect sizes. Fourth, a considerable number of participants in the sample exhibited low values of both REM and NREM variables, including zero duration, suggesting potential limitations in the algorithm’s ability to sensitively capture certain sleep patterns. Furthermore, the sample consisting only of employees from a single Austrian company may not fully represent broader population with diverse demographics and occupational profiles. This homogeneity of the sample may restrict the applicability of our results to populations with different stress levels, cultural backgrounds, or health behavior. Future research should aim to replicate these findings in more diverse and representative samples to enhance generalizability. Moving forward, improvement of the algorithm, such as incorporating the assessment of bodily movement, appears to be crucial to enhance its accuracy and validity.

## Conclusions and practical implications

Taken together, the application of the SleepECG algorithm in assessing the relationship between lifestyle factors and sleep quality yielded valuable insights. Our findings confirmed significant associations between sleep duration and both REM and NREM sleep stages, highlighting the importance of considering not only sleep quantity but also sleep architecture in evaluating overall sleep health. Results suggests that moderate PA is positively linked to improve REM and NRWM sleep stages, indicating its potential to enhance sleep quality. It also highlights the significant association between fruit and vegetable consumption and various sleep quality indicators, emphasizing the potential impact of dietary habits on sleep outcomes. Additionally, it points out the negative correlation between BMI and sleep quality, specifically relating higher BMI to poorer sleep quality, particularly a decrease in REM sleep stages. These findings emphasize the potential influence of lifestyle factors on sleep quality (Vargas et al. 2014; Krističević et al. 2018; Wang and Boros 2021

Together, these findings contribute to the growing body of evidence supporting the contribution of lifestyle behaviors to sleep quality (Wakasugi et al. 2014; Dzierzewski et al. 2021; Lee et al. 2021). The first application of the SleepECG algorithm in quantifying REM and NREM sleep stages in ambulatory settings underscores its use as a valuable tool for gathering nuanced data on sleep parameters in an individual’s natural setting. Although promising, there is need for further refinement and validation of the algorithm to enhance its accuracy and validity.

## Data Availability

Data are available from the author upon request.
